# Scalable Multilayer PTFE‐Reinforced Membranes for Durable, High‐Current‐Density Hydroxide Exchange Membrane Water Electrolysis in Dilute Alkali

**DOI:** 10.1002/advs.77388

**Published:** 2026-08-24

**Authors:** Zhiwei Ren, Yun Zhao, Rui Zhang, Tao Wei, Yingran Zhu, Yangkai Han, Tongzhou Li, Haitao Zhang, Zhigang Shao

**Affiliations:** ^1^ Fuel Cell System and Engineering Laboratory Key Laboratory of Fuel Cells & Hybrid Power Sources Dalian Institute of Chemical Physics Chinese Academy of Sciences Dalian China; ^2^ University of Chinese Academy of Sciences Beijing China; ^3^ Liaoning Binhai Laboratory Dalian Liaoning China

**Keywords:** dilute alkalis, hydroxide exchange membrane water electrolysers, interfacial conformability, large‐scale

## Abstract

Hydroxide exchange membrane water electrolysis (HEMEL) is a promising route to cost‐effective green hydrogen using renewable energy, yet its commercial potential is hindered by insufficient performance and durability. Here, we address this challenge with an ultrathin, multilayer membrane design that integrates polytetrafluoroethylene (PTFE) reinforcement with low glass‐transition temperature (T_g_) polymers. Fabricated via a scalable hot‐pressing process, these membranes (QPS‐PTFE3‐nL, n = 3, 4, 5) exhibit anisotropic hydration, alkali stability, and favorable interfacial resistance. This integrated architecture enables stable operation in a 0.1 M KOH dilute electrolyte. The HEMEL employing the optimized QPS‐PTFE3‐5L membrane achieves an outstanding current density of 3.7 A cm^−2^ at 1.8 V and demonstrates remarkable durability, operating continuously at 2.0 A cm^−2^ for over 3300 h—representing state‐of‐the‐art performance in this field. Post‐test analysis reveals that performance decay primarily stems from cathode catalyst layer degradation rather than membrane failure, underscoring the robustness of the membrane design. The scalability of this approach is validated by a 160 cm^2^ cell that maintains stable operation for 52 days under intermittent cycling. This work provides a straightforward and scalable membrane strategy to realize durable, high‐performance HEMEL systems.

## Graphical abstract

Low‐T_g_ HEMs are designed to improve membrane‐electrode adhesion by promoting intimate contact with catalyst layers. After hydration, the membrane develops a hydrogel‐like network that supports water uptake and ion transport while retaining mechanical robustness. Together with superior chemical stability, these features enable efficient and durable hydroxide exchange membrane water electrolysis.

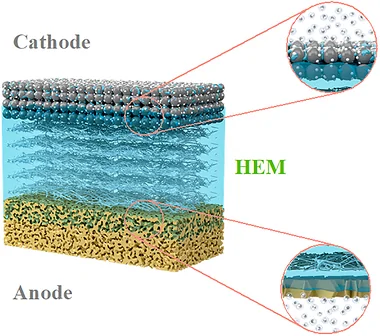

## 1 Introduction

Hydrogen has emerged as a strategic energy carrier essential for achieving carbon neutrality, driving urgent demand for efficient, scalable, and low‐cost water electrolysis technologies [1, 2, 3, 4]. Hydroxide exchange membrane water electrolysis (HEMEL) is a highly promising route, as its alkaline operation enables the use of non‐precious metal catalysts and offers compatibility with intermittent renewable electricity [5]. However, its practical adoption remains hampered by inadequate performance and durability, with persistent bottlenecks centered on the hydroxide exchange membrane (HEM) and its interface within the membrane‐electrode assembly (MEA). Therefore, improving HEMEL durability requires not only chemically robust membrane materials, but also a clear understanding of how membrane degradation couples with interfacial failure under operating conditions.

The HEM exerts a first‐order influence on HEMEL lifetime. A primary failure mechanism originates from the intrinsic chemical vulnerability of cationic groups under concentrated alkaline conditions [6, 7, 8, 9]. Operating in dilute electrolyte (e.g., 0.1 m KOH) offers a compelling mitigation strategy, suppressing chemical degradation by orders of magnitude while conferring system‐level advantages like reduced corrosion and mitigated catalyst deactivation risks during electrode reversal [10, 11]. Nevertheless, shifting from concentrated to dilute alkaline operation does not eliminate durability limitations; rather, it transfers a substantial portion of the stability burden from intrinsic chemical resistance to water management, dimensional control, and interfacial integrity. In particular, reduced alkalinity intensifies interfacial and mechanical challenges, as hydration‐driven membrane swelling imposes stringent new demands on the mechanical robustness and interfacial stability of the MEA [12, 13].

Consequently, the MEA interface becomes a decisive durability bottleneck, where membrane swelling, ion transport, and mechanical adhesion are strongly coupled. This brings the MEA interface into sharp focus as the functional and often weakest locus in HEMELs, governing both performance and durability [14]. While a robust interface is essential to minimize ohmic loss and sustain efficient mass transport at high current densities, it is highly vulnerable [15, 16]. The combined effects of a local alkaline environment and cyclic hydration‐swelling stresses promote progressive interfacial degradation through delamination and ionomer leaching [17, 18]. This initiates a self‐accelerating failure loop, explaining why stable operation beyond 2000 h at 2 A cm^−2^ remains a significant challenge [6].

In this context, strengthening interfacial cohesion must be considered together with membrane dimensional stability, rather than as an isolated interface‐engineering problem. Existing strategies have focused on modifying the catalyst layer ionomer or the membrane itself. For instance, covalent crosslinking between the catalyst layer and membrane has been shown to enhance stability [19, 20]. The membrane surface properties are equally vital. Many state‐of‐the‐art poly(aryl)‐based HEMs possess high T_g_ (>200°C), which hinders the formation of an intimate, adhesive interface during standard MEA fabrication [21]. We previously addressed interfacial adhesion by developing low‐T_g_ polystyrene (PS)‐based HEMs reinforced with a two‐dimensional mesh [22]. While this improved interfacial contact, managing severe through‐plane swelling of the polymer phase during operation remains challenging, often leading to local polymer delamination from the support mesh. This persistent failure mode highlights the need for an integrated design that simultaneously ensures dimensional stability, efficient ion transport, and robust interfacial adhesion.

To mitigate swelling, three‐dimensional reinforcement using scaffolds such as expanded polytetrafluoroethylene (PTFE) has become prevalent [23, 24, 25]. Although PTFE reinforcement effectively curbs dimensional change, conventional single‐layer substrates often yield membranes with insufficient mechanical strength–‐a key property for withstanding the cyclic swelling‐shrinkage stresses during long‐term electrolysis and achieving extended HEMEL lifetime [26]. Therefore, a rational reinforcement architecture should go beyond simple swelling suppression and provide continuous mechanical support throughout the membrane thickness, while preserving ion‐conducting pathways and enabling strong membrane‐electrode interfacial contact.

Herein, we report a synergistic membrane strategy that concurrently tackles the intertwined challenges of membrane swelling, mechanical stress, and interfacial adhesion for dilute‐alkali operation. We developed a chemically stable, low‐T_g_ quaternized polystyrene (QPS) polymer and integrated it with an ultrathin PTFE (3 µm) scaffold to fabricate a reinforced elementary membrane. Scalable multilayer membranes (QPS‐PTFE3‐nL, n = 3, 4, 5) were then constructed via a hot‐pressing process. This layer‐by‐layer assembly significantly improves mechanical properties while creating an optimized, low‐resistance MEA interface. The resulting membranes exhibit a unique hydration behavior–‐combining high water uptake with strongly anisotropic swelling–‐and exceptional alkali stability. When integrated into a HEMEL operating with 0.1 m KOH, the optimized QPS‐PTFE3‐5L membrane enables outstanding performance (3.7 A cm^−2^ at 1.8 V) and remarkable durability, operating for over 3300 h at 2.0 A cm^−2^. Post‐test analysis confirms the membrane's robustness, identifying cathode catalyst layer degradation as the primary failure reason. Furthermore, the scalability of this approach is demonstrated by a 160 cm^2^ cell that operates stably for 52 days.

## 2 Results and Discussion

### 2.1 Synthesis and Characterization of Multilayer QPS‐PTFE3‐nL HEMs

**FIGURE 1:**
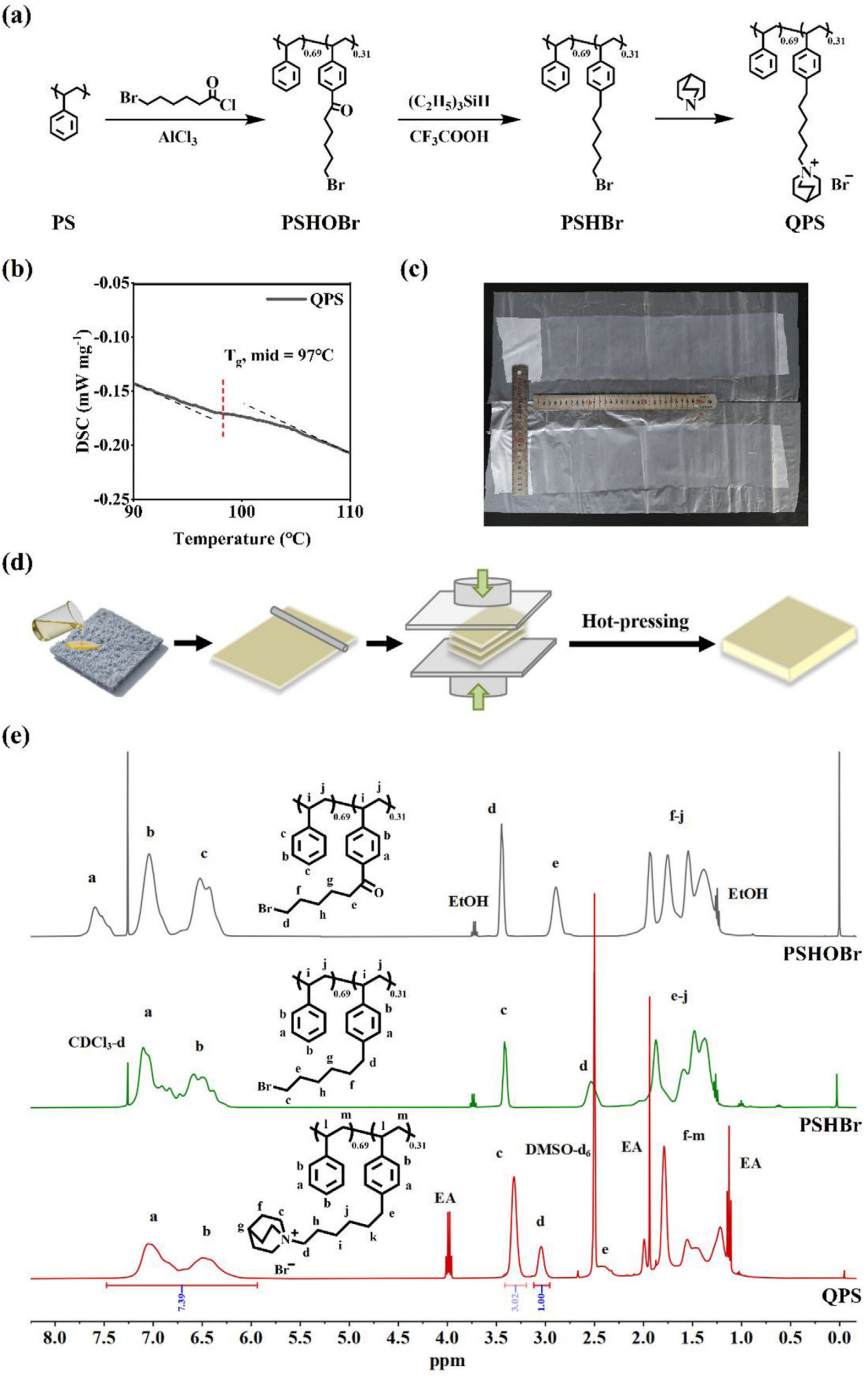
(a) Synthetic route of QPS. (b) DSC showing the Tg of QPS. (c) Optical image of QPS‐PTFE3‐1L. (d) Schematic of the layer‐by‐layer hot‐pressing fabrication of QPS‐PTFE3‐nL (n = 3, 4, 5). (e) ^1^H NMR spectra of the polymer intermediates (PSHOBr, PSHBr) and final product (QPS) in d‐solvents with 5% trifluoroacetic acid as a co‐solvent to suppress the water peak at 3.33 ppm. (EtOH: ethanol; EA: ethyl acetate).

To realize a low T_g_ membrane polymer, we selected PS as a modifiable backbone. The synthetic pathway is illustrated in Figure 1a. Following a modified literature procedure, a 6‐bromohexanoyl group was first introduced at the para‐position of the benzene ring via Friedel‐Crafts acylation, yielding the intermediate PSHOBr (Figure 1a) [27]. Successful formation was confirmed by ^1^H NMR (Figure 1e), which showed the characteristic downfield shift of the aromatic H ortho to the electro‐withdrawing carbonyl group (7.6 ppm, labeled a), alongside distinct signals for the aliphatic chain protons.

Subsequent reduction of the carbonyl group using trifluoroacetic acid and triethylsilane produced PSHBr, as evidenced by the disappearance of the aromatic signal at 7.6 ppm and the appearance of a new peak at 2.53 ppm (labeled d, Figure 1e). Finally, a nucleophilic substitution reaction with quinuclidine yielded the target quaternized polystyrene (QPS). The ^1^H NMR spectrum of QPS (Figure 1e) displayed the diagnostic resonances of the quinuclidinium cation (3.3 ppm, labeled c) and the adjacent tether methylene group (3.0 ppm, labeled d). Integration confirmed a high cation grafting ratio of 31%.

Differential scanning calorimetry (DSC) revealed a relatively low T_g_ for QPS, with T_g, mid_ determined to be 97°C from the midpoint of the baseline shift in the curve (Figure 1b), which is significantly lower than that of typical polyaryl‐based polymers. This low T_g_, coupled with the polymer's solubility in ethanol, facilitates straightforward membrane processing at moderate temperatures. Although the low T_g_ polymer is beneficial for improving membrane‐catalyst layer adhesion, its severe swelling under hydrated alkaline conditions would compromise dimensional stability and ion transport continuity. Therefore, structural reinforcement is necessary to retain its interfacial advantage while suppressing swelling‐induced deformation. An initial single‐layer composite membrane, QPS‐PTFE3‐1L, was prepared via blade‐coating using a 3 µm ultrathin PTFE substrate as a reinforcing scaffold (Figure S1). Scalable multilayer membranes, QPS‐PTFE3‐nL (n = 3, 4, 5), were then prepared through hot‐pressing of the corresponding number of QPS‐PTFE3‐1L layers (Figure 1c,d).

### 2.2 Morphology of the QPS‐PTFE3‐nL HEMs

**FIGURE 2:**
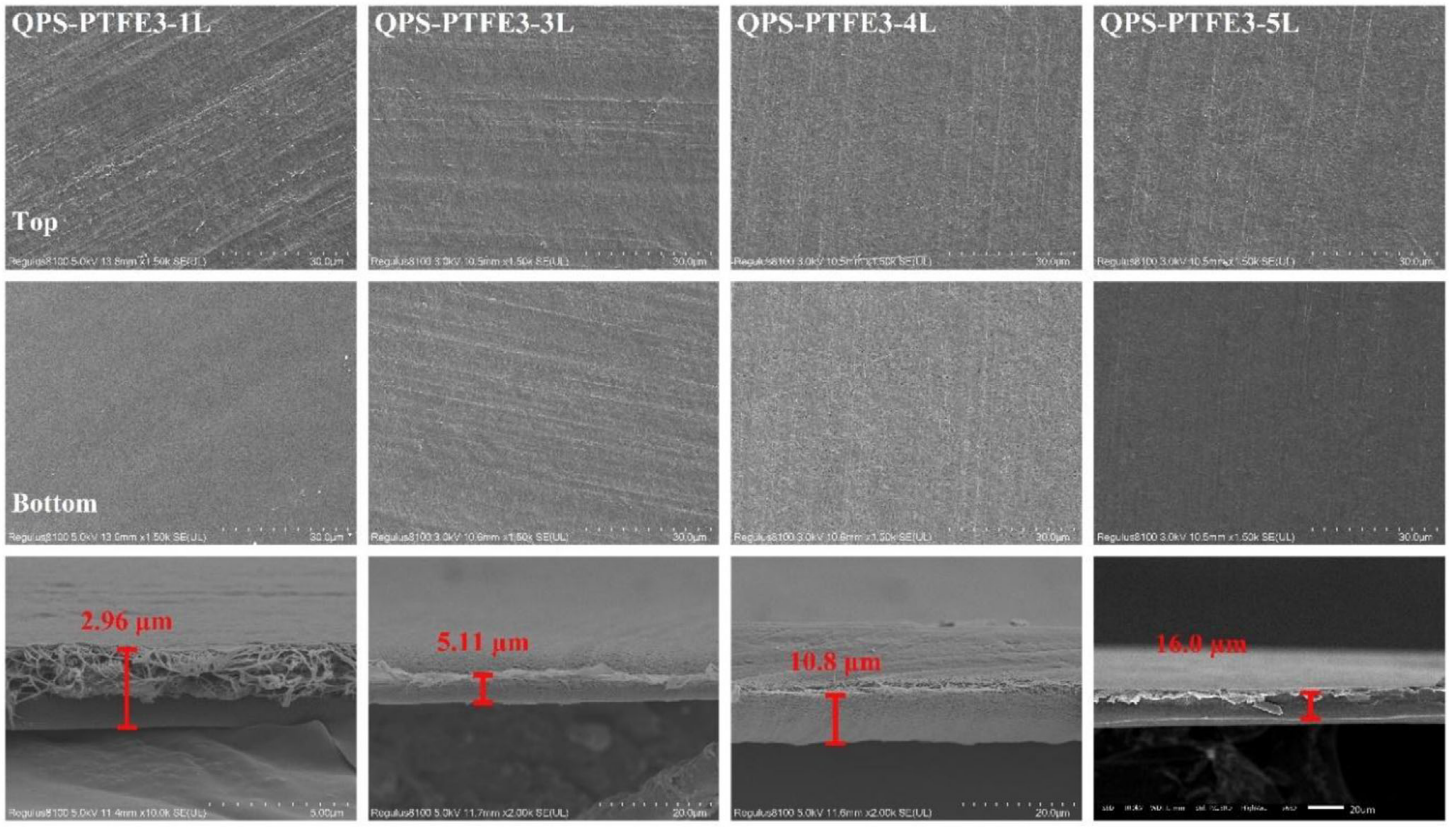
SEM images of membrane surface and cross‐sections for QPS‐PTFE3‐nL (n = 1, 3, 4, 5).

The morphology of QPS‐PTFE3‐nL HEMs was characterized by scanning electron microscopy (SEM). As shown in Figure 2, both surfaces of the QPS‐PTFE3‐1L appear dense and uniform. The cross‐sectional image confirms successful compositing, clearly revealing the PTFE reinforcement film embedded within a total membrane thickness of ∼3 µm. Multilayer membranes (n = 3, 4, 5) were subsequently fabricated by hot‐pressing the single‐layer precursors above the T_g_ of QPS. To further verify the structural integrity of the multilayer architecture, five PTFE substrates were subjected to the same hot‐pressing process. No obvious delamination or separated layered structure was observed in the cross‐sectional SEM image of the hot‐pressed five‐layered resin‐unfilled PTFE substrates, supporting the effectiveness of the hot‐pressing process in consolidating the multilayer scaffold (Figure S2). Consistently, the cross‐sectional SEM images of QPS‐PTFE3‐nL membranes (Figure 2) show that the individual QPS‐PTFE layers are well consolidated after hot‐pressing, with no obvious delamination, voids, or poorly bonded interlayers. This indicates good interfacial adhesion and favorable polymer fluidity during processing. Therefore, the interlayer regions are unlikely to act as additional ion transport barriers, but instead contribute to the integrated mechanical confinement of the membranes. The measured thicknesses are lower than the theoretical sum of their constituent layers, attributable to further densification under a high applied pressure (3 MPa) during hot‐pressing. To further evaluate the interfacial integrity of the hot‐pressed multilayer membrane, wet‐state H_2_ permeability was measured using commercial PiperION A20 as a benchmark. As shown in Figure S3, the H_2_ permeability of QPS‐PTFE3‐5L is approximately 1.5 times that of PiperION A20, and no leakage‐like behavior was observed. These results confirm the successful fabrication of integrated, defect‐free multilayer HEMs.

### 2.3 Physicochemical Properties of QPS and QPS‐PTFE3‐nL HEMs

**FIGURE 3:**
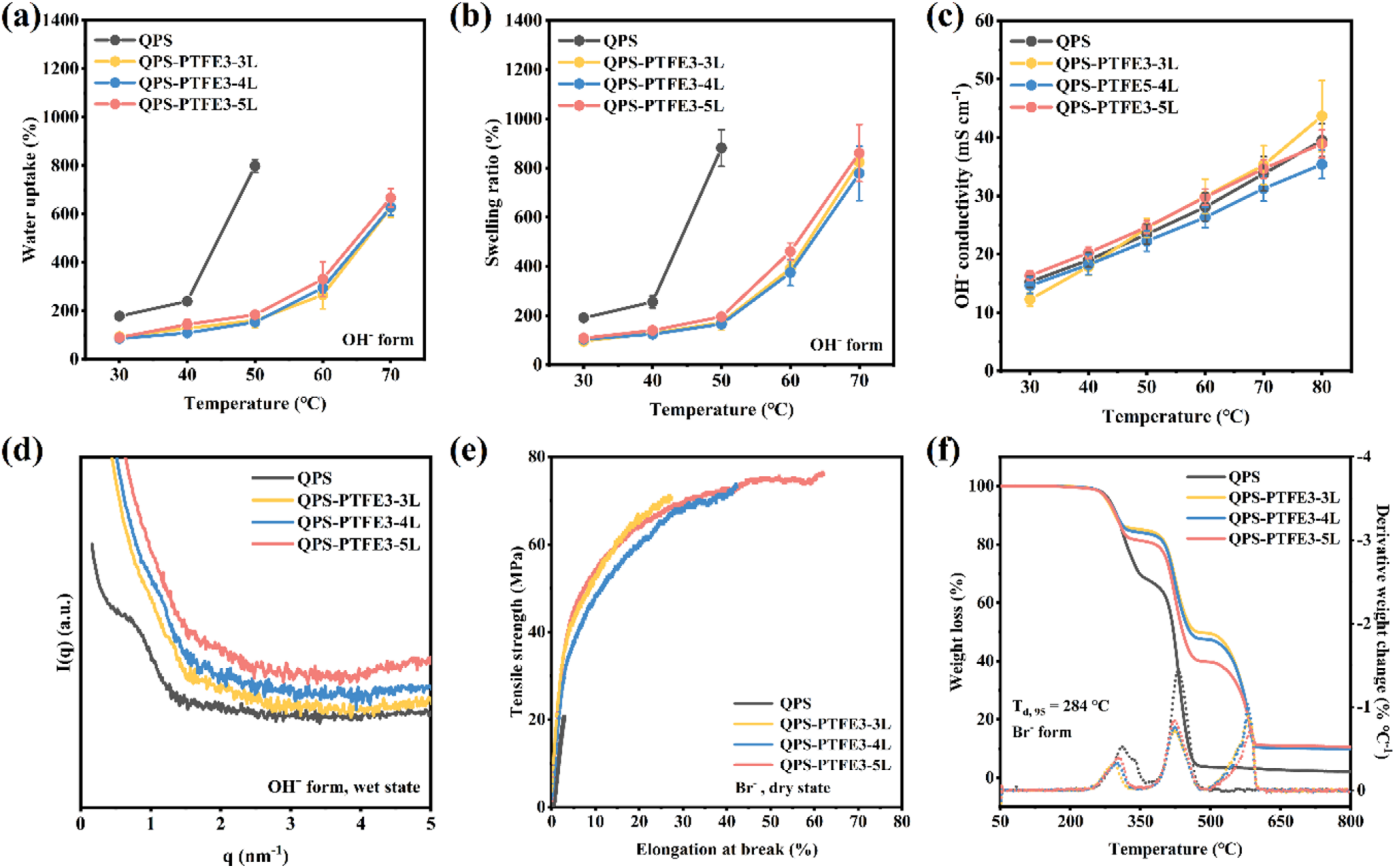
Physicochemical properties of QPS and QPS‐PTFE3‐nL (n = 3, 4, 5) HEMs. (a) Water uptake, (b) Swelling ratio and (c) OH^−^ conductivity as a function of temperature. (d) SAXS spectra, (e) Mechanical properties and (f) Thermal degradation profiles from TGA/DTG.

Water uptake and dimensional swelling are critical determinants of membrane stability. As shown in Figure 3a, pristine QPS exhibits excessive, disruptive hydration at 50°C, absorbing ∼800% water by mass. This extreme swelling, which renders the membrane a soft gel at higher temperatures, results from water‐induced plasticization overwhelming the polymer's intrinsic T_g_. In contrast, the PTFE‐reinforced multilayer membranes, QPS‐PTFE3‐nL (n = 3, 4, 5), show markedly suppressed water uptake (∼150% at 50°C) and retain structural integrity up to 70°C (Figure 3b). The PTFE scaffold imposes a strong anisotropic constraint on membrane swelling. Although through‐plane swelling remains appreciable, the in‐plane swelling of QPS‐PTFE3‐nL is effectively restricted to below 10% (Figure S4). Such anisotropic dimensional response is beneficial for electrolysis operation because in‐plane expansion more directly affects dimensional matching with the catalyst layer and membrane‐electrode interfacial integrity, whereas through‐plane swelling can be mechanically confined in the assembled cell. In particular, compared with PiperION A20, the higher through‐plane swelling of QPS‐PTFE3‐nL may help fill interfacial gaps under cell compression, thereby contributing to improved membrane‐catalyst layer contact [28, 29]. However, since composite membranes undergo severe swelling above 50°C, which leads to thermal softening, 50°C is determined as the reliable operating temperature. Interestingly, above 50°C, the in‐plane swelling of the composite membranes slightly decreases, due to the onset of the hydrated polymer's T_g_ and competing strain from pronounced through‐plane expansion (Figure S4b). Critically, despite this substantial hydration, no interlayer delamination is observed, confirming that the hot‐pressing process creates an interlocked, cohesive multilayer architecture. This unique behavior–‐high water uptake coupled with restrained in‐plane swelling–‐forms a stable hydrogel‐like matrix that facilitates rapid water transport while maintaining mechanical integrity.

The hydroxide conductivity of the membranes was evaluated using a four‐probe method (Figure 3c). Despite its high ion exchange capacity (IEC), pristine QPS shows poor OH^−^ conductivity due to excessive water uptake diluting the cationic charge carriers. The composite membranes, while benefiting from controlled swelling, exhibit a proportionally lower IEC (Table 1), resulting in similarly moderate hydroxide conductivities. For comparison, the OH^−^ conductivity of commercial PiperION A20 was also measured under the same protocol. As shown in Figure S5, PiperION A20 exhibits substantially higher OH^−^ conductivity than QPS‐PTFE3‐nL over the tested temperature range. This result indicates that QPS‐PTFE3‐nL is not designed to maximize intrinsic ion conductivity. Instead, its design emphasizes the balance between strong hydrophilicity, low‐T_g_‐assisted interfacial adhesion, and PTFE‐reinforced mechanical integrity. Small‐angle x‐ray scattering (SAXS) analysis of the hydrated composite membranes reveals no distinct scattering peaks (Figure 3d), suggesting that the hydrophobic PTFE network disrupts the formation of long‐range, ordered ionic channels, which may contribute to the observed conductivity.

**TABLE 1:** Fundamental properties of the HEMs.

HEM	IEC^a^, (mmol g^−1^)	σ^b^, (mS cm^−1^)		WU^c^, (%)		SR^d^, (%)		TS^e^, (MPa)	EB^f^, (%)
	Titra.	30°C	80°C	30°C	70°C	I‐P	T‐P	Br^−^, dry	
QPS	1.62	15.3	39.5	178.3	N/A	237.6	129.5	20.6	2.9
QPS‐PTFE3‐3L	1.08	12.2	43.7	93.5	626.2	107.9	181.7	70.7	27.4
QPS‐PTFE3‐4L	1.08	14.6	35.4	85.4	628.1	109.9	185.4	73.1	42.2
QPS‐PTFE3‐5L	1.07	16.3	38.9	89.6	666.6	108.5	191.3	75.9	62.0

^a^ The IEC was measured by titration in Cl^−^ form.

^b^ Measured under the fully hydrated state in OH^−^ form.

^c^ Measured under the fully hydrated state in OH^−^ form.

^d^ Measured under the fully hydrated state in OH^−^ form (I‐P: in‐plane, T‐P: through‐plane).

^e,f^ Measured under the dry state in Br^−^ form.

The reinforcement strategy substantially enhances mechanical performance (Figure 3e). The tensile strength increases from 20.6 MPa for pristine QPS to 75.9 MPa for QPS‐PTFE3‐5L, while the strain at break rises from 2.9% to 62.0%. This enhancement is attributed to the formation of a three‐dimensional interpenetrating network during hot‐pressing, where the low T_g_ QPS flows to entangle molecular chains across layers, supported by the multilayer PTFE scaffold. Thermogravimetric analysis (TGA) confirms the thermal stability of QPS‐PTFE3‐nL (n = 3, 4, 5) HEMs, with a 95% weight loss temperature (T_d, 95_) of 284°C (Figure 3f) [8]. The derivative weight‐loss curve shows characteristic degradation steps: cation decomposition near 305°C, polymer backbone degradation around 425°C, and PTFE decomposition at approximately 578°C. Consequently, the improved interfacial adhesion, suppressed swelling, and stabilized ion‐transport pathways collectively contribute to the enhanced electrolysis performance and durability of QPS‐PTFE3‐nL.

### 2.4 Alkali Stability of QPS‐PTFE3‐nL (n = 3, 4, 5) HEMs

**FIGURE 4:**
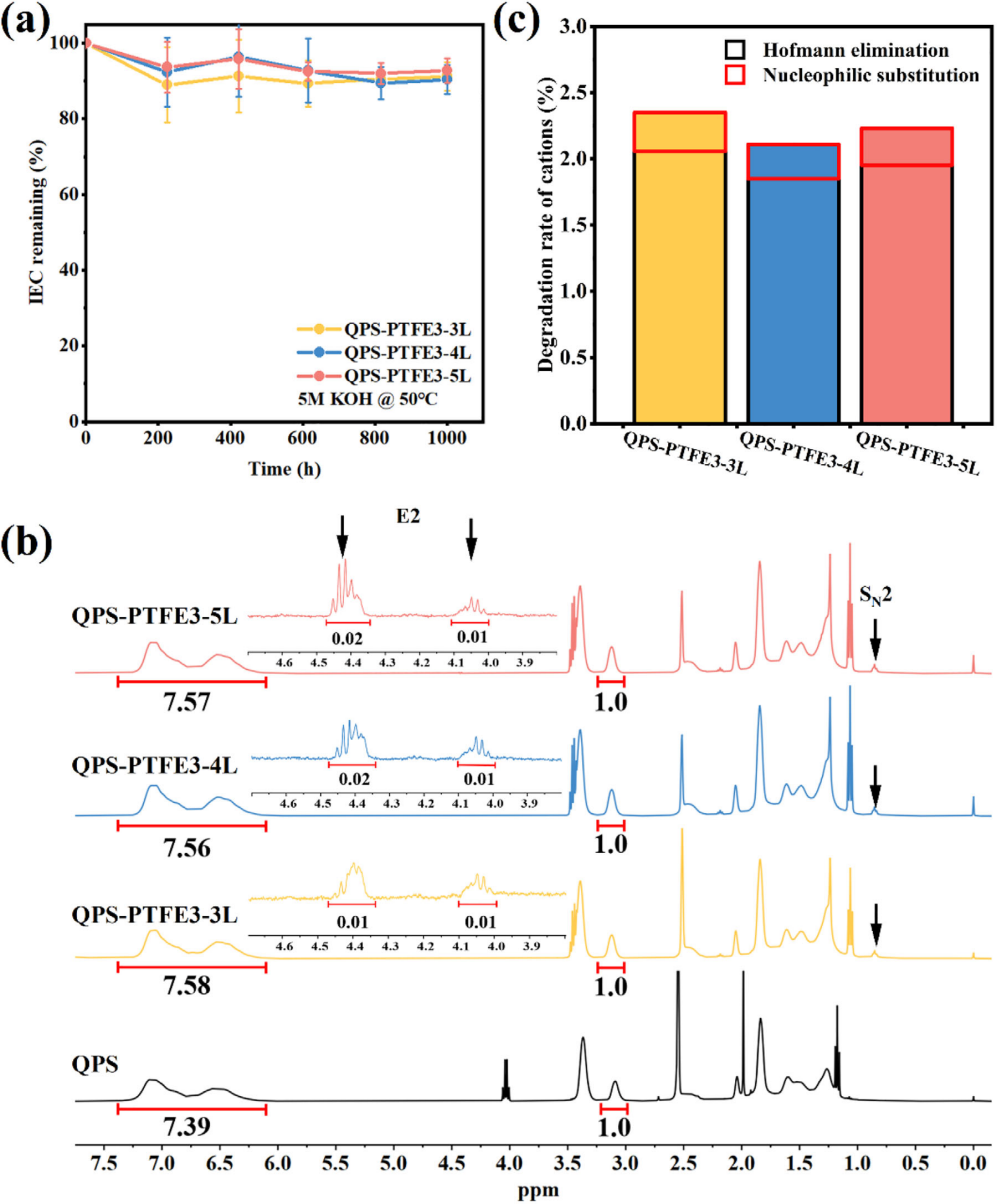
Alkaline stability evaluation of QPS‐PTFE3‐nL (n = 3, 4, 5) HEMs. (a) Retention of IEC over time during immersion in 5 m KOH at 50°C. (b) ^1^H NMR spectra of the membranes after alkaline treatment, indicating structural integrity. (c) Quantitative analysis of total ionic loss and the contribution of specific degradation pathways (Hofmann elimination vs. nucleophilic substitution) derived from ^1^H NMR spectra.

Alkali stability is a key factor of the operational lifetime for HEMs [30]. To evaluate this property under accelerated conditions, HEMs were immersed in 5 m KOH at 50°C. The alkali stability was monitored by tracking the IEC over time (Figure 4a). The QPS‐PTFE3‐nL (n = 3, 4, 5) HEMs, sharing the same polymer, exhibited identical degradation profiles, retaining approximately 90% of their initial IEC after 1000 h–‐demonstrating excellent alkali resistance. Structural changes post‐degradation were characterized by ^1^H NMR spectroscopy (Figure 4b). The spectra indicate that the degradation proceeds primarily via two pathways: nucleophilic substitution (S_N_2) and Hofmann elimination (E2) (Figure S6) [31]. By quantifying the loss of characteristic proton signals relative to stable aromatic references, the extent and pathway of ionic loss were estimated (Figure 4c). The membranes experienced a total ionic loss of ∼2.2%, with E2 accounting for about 85% of the degradation.

In addition, complementary stability was assessed by measuring the change in HCO_3_^−^ conductivity over time (Figure S7a). Despite high cation retention, the HCO_3_^−^ conductivity dropped to about 35% of its initial value after 1000 h. This discrepancy is attributed to dimensional instability rather than chemical degradation. As shown in Figure S7b, the membrane thickness nearly doubled during the test, diluting the fixed cation concentration and thereby reducing conductivity. This pronounced and continuous swelling arises from the test protocol, which involves alternating immersion in alkaline solution and pure water. When transferred to water, a high osmotic pressure gradient drives water into the membrane. This water acts as a plasticizer, increasing polymer chain mobility and lowering the hydrated membranes' T_g_. The reduced T_g_, in turn, facilitates further water uptake, establishing a self‐accelerating cycle of hydration and swelling that compromises dimensional integrity under extended immersion.

### 2.5 Performance and Durability of the HEMEL

The electrolysis performance of the QPS‐PTFE3‐nL membrane was evaluated in a HEMEL with a γ‐Ni(Fe)OOH@NF anode and a PtRu/C cathode [32]. To identify an appropriate multilayer configuration for HEMEL operation, QPS‐PTFE3‐nL HEMs with different layer numbers were systematically screened (Figure S8). QPS‐PTFE3‐3L suffered from short‐circuiting during cell assembly, whereas QPS‐PTFE3‐4L exhibited unstable cell voltage, indicating insufficient electrical insulation for reliable operation. In contrast, QPS‐PTFE3‐5L, QPS‐PTFE3‐7L, QPS‐PTFE3‐9L, and QPS‐PTFE3‐11L enabled stable cell operation. Among these stable configurations, QPS‐PTFE3‐5L exhibited the highest current density in both 0.1 and 1.0 m KOH and was therefore identified as the optimal multilayer configuration within the investigated range.

**FIGURE 5:**
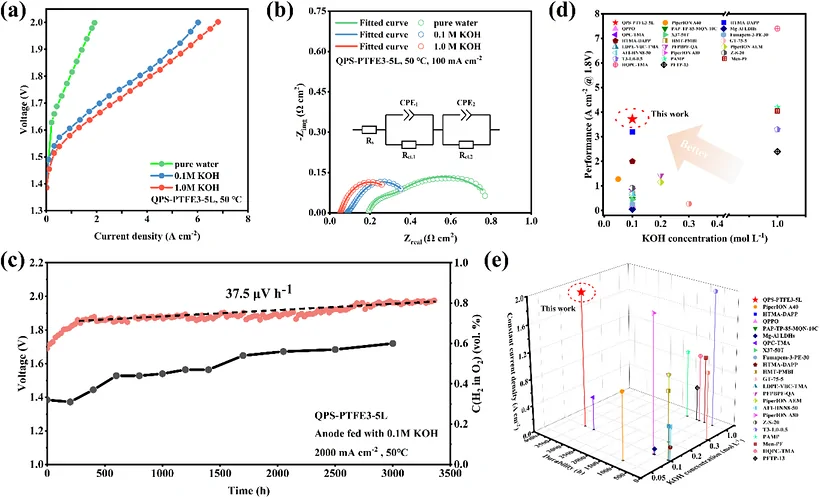
The performance and durability of the HEMEL with QPS‐PTFE3‐5L HEM. (a) Polarization curves and (b) corresponding Nyquist plots measured with different electrolyte feeds: 1.0 m KOH, 0.1 m KOH, and pure water. (c) Long‐term stability test at a constant current density of 2000 mA cm^−2^, using 0.1 m KOH at 50°C. Benchmarking of this work against prior studies in terms of (d) peak current density at 1.8 V and (e) operational lifetime (see Table S3).

Based on this screening, QPS‐PTFE3‐5L was selected for further evaluation under different electrolyte conditions. Its electrolysis performance was sequentially tested under pure water, 0.1 m KOH, and 1.0 m KOH feed conditions (Figure 5a). As expected, switching from pure water to alkaline electrolyte significantly enhanced performance due to improved electrode kinetics and ion conduction [33]. Notably, when reducing the KOH concentration from 1.0 to 0.1 m, the current density at 1.8 V decreased only slightly, from 4100 to 3700 mA cm^−2^. This limited performance loss under dilute alkali is attributed to the improved interfacial contact achieved via the low T_g_ of QPS polymer, which forms a stable, adhesive interface with the catalyst layers during hot‐pressing (Figure S9). At high current densities (>5000 mA cm^−2^), operation with 0.1 m KOH induces mass transport polarization due to the limited hydroxide ion flux. Further analysis of the EIS data reveals that when the feed was changed from pure water to KOH solution, both the ohmic resistance and the low‐frequency interfacial/transport resistance decreased markedly (Figure 5b and Table S1). Specifically, R_s_ decreased from 191.1 mΩ cm^2^ in pure water to 87.2 and 52.3 mΩ cm^2^ in 0.1 and 1.0 m KOH, respectively, indicating enhanced ionic conduction in the cell [34]. Meanwhile, R_ct,2_ decreased from 601.7 to 303.7 and 200.4 mΩ cm^2^, suggesting that KOH feeding effectively alleviated low‐frequency interfacial and ion‐transport limitations [35]. This improvement is mainly attributed to the enhanced electrolyte conductivity, increased OH^−^ availability in the catalyst layer, and facilitated ion transport across the membrane‐electrode interface.

To further evaluate whether the lower ex situ membrane conductivity limits practical HEMEL performance, a side‐by‐side cell benchmark was performed using commercial PiperION A20 under identical conditions. As shown in Figure S10a, QPS‐PTFE3‐5L exhibits competitive HEMEL performance and reaches a higher current‐density region than PiperION A20 at 60°C in 0.1 m KOH. This high current‐density advantage is consistent with its lower resistance of 0.0671 Ω cm^2^, compared with 0.0765 Ω cm^2^ for the PiperION A20‐based cell, indicating more efficient ionic conduction through the membrane and across the membrane‐electrode interface (Figure S10b and Table S2). As discussed above, QPS‐PTFE3‐5L achieves a favorable balance between sufficient water uptake and limited in‐plane swelling. Its sufficient water uptake maintains the hydrated ionic domains required for OH^−^ conduction. Meanwhile, the relatively low‐T_g_ QPS polymer promotes conformal contact with the catalyst layer, reducing interfacial voids and discontinuities in the ionic pathways. The multilayer PTFE reinforcement further suppresses excessive in‐plane swelling, thereby limiting dimensional mismatch and preserving membrane‐electrode contact during hydration and high current‐density operation. These properties collectively maintain continuous OH^−^ transport pathways within the membrane and across the membrane‐electrode contact region. Beacuse the local temperature of the electrolyzer may exceed the recommended operating temperature of 50°C under high‐current‐density operation, the H_2_ concentration in the O_2_ outlet was also monitored at 60°C across different current densities. As shown in Figure S10a, QPS‐PTFE3‐5L exhibits higher H_2_ crossover than commercial PiperION A20, but the H_2_ concentration remains below the safety threshold, confirming its operational safety in the assembled MEA. These results suggest that although the membrane swells significantly above 50°C, the mechanical constraint of the cell effectively limits excessive expansion and maintains sufficient gas‐barrier capability for safe operation under the tested conditions.

The long‐term durability of the HEMEL was assessed under dilute alkaline conditions. Operating at a constant current density of 2000 mA cm^−2^ with 0.1 m KOH feed at 50°C, the cell demonstrated exceptional stability for 3360 h with a low voltage decay rate of 37.5 µV h^−1^ (Figure 5c). This unprecedented durability is attributed to the synergistic combination of a robust MEA interface, the membrane's high water uptake (ensuring sufficient hydration at the cathode), and its excellent chemical stability. Moreover, the improved durability further confirms that the multilayer architecture effectively mitigates swelling‐induced structural instability during long‐term electrolysis. Operational safety was concurrently verified by monitoring H_2_ crossover. The H_2_ content in the O_2_ remained below 0.7 vol.% throughout the test, indicating that QPS‐PTFE3‐5L provided sufficient gas‐separation capability to ensure safe operation under the tested conditions. Inital increases in voltage and crossover were observed within the first 500 h, after which both stabilized, indicating the establishment of a steady operational state. As summarized in Figure 5d,e and Table S3, the performance and durability of our QPS‐PTFE3‐5L‐based HEMEL in 0.1 m KOH surpass most reported systems, including many operating under more concentrated (1.0 m KOH) conditions. To our knowledge, this work sets new benchmarks for both current density and long‐term durability in HEMELs.

**FIGURE 6:**
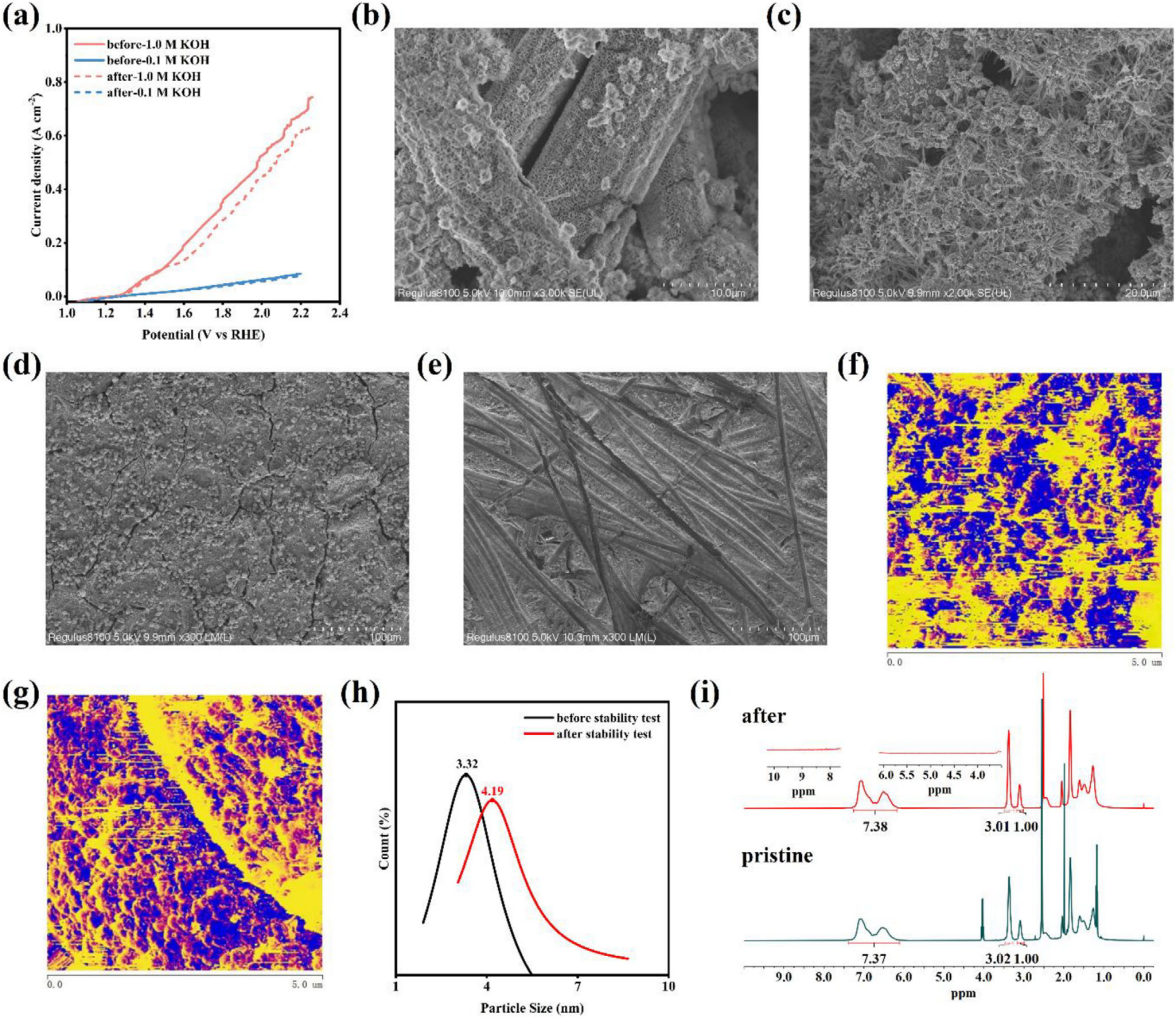
Post‐mortem analysis of the HEMEL MEA. (a) LSV curves of pristine and aged γ‐Ni(Fe)OOH@NF at 25°C. Morphology images of (b) pristine and (c) aged γ‐Ni(Fe)OOH@NF. Surface morphology images of (d) pristine and (e) aged CCL. AFM images of (f) pristine and (g) aged CCL. (h) The pristine and aged PtRu particle size distribution (counted from 100 particles). (i) ^1^H NMR spectra of pristine and aged QPS‐PTFE3‐5L HEMs.

Post‐mortem analysis was conducted to investigate the HEMEL components after the 3360 h durability test. The γ‐Ni(Fe)OOH@NF anode was carefully separated from the MEA for analysis. Linear sweep voltammetry (LSV) showed that the aged γ‐Ni(Fe)OOH@NF catalyst retained nearly identical oxygen evolution reaction activity to the pristine sample in 0.1 m KOH, with only minor discrepancies in 1.0 m KOH (Figure 6a), indicating negligible catalytic degradation. However, morphological change was observed via SEM: the pristine nanosheet structure evolved into a nanowire‐like morphology with partial exposure of the nickel foam substrate (Figures 6b,c and S11). While energy dispersive x‐ray spectroscopy (EDS) confirmed uniform distribution of Ni, Fe, and O (Figure S13), the Fe content in the aged anode decreased by approximately 50% (Figure S12 and Table S4), indicating continuous Fe leaching during operation [36, 37, 38]. X‐ray photoelectron spectroscopy (XPS) revealed unchanged valence states for Ni and Fe but an increased abundance of M‐O species, consistent with a high‐potential oxidative environment (Figure S14) [39]. Meanwhile, the observed variations in elemental content were in excellent agreement with the findings from EDS measurements (Table S5).

In contrast, the cathode catalyst layer (CCL) underwent significant morphological and structural changes. SEM imaging revealed that the pristine CCL had a smooth, porous surface (Figures 6d and S15a), whereas the aged CCL appeared denser, with surface indentations from the porous transport layer (PTL) and a significant loss of pore structure (Figures 6e and S15b). Atomic force microscopy (AFM) phase imaging showed a uniformly interconnected morphology of catalyst (blue‐purple) and ionomer (yellow) phases in the pristine CCL (Figure 6f). After aging, severe phase separation and agglomeration were evident, disrupting the integrated ionomer‐catalyst network (Figures 6g and S16). This densification and phase segregation synergistically degrade cathode performance by: (i) collapsing pore structures and blocking mass transport pathways for H_2_O and H_2_ [40, 41, 42, 43]; (ii) disrupting the ion‐conduction network, creating dead zones and inhomogeneous current distribution [19, 44, 45]; and (iii) increasing interfacial resistance with the PTL, accelerating localized corrosion [46, 47, 48, 49].

High‐resolution TEM of the isolated PtRu/C catalyst confirmed nanoparticle aggregation after testing (Figure S17). Particle size analysis showed an increase in PtRu nanoparticle size after operation, indicating a loss of electrochemically active surface area caused by dissolution/redeposition, Ostwald ripening, and particle detachment induced by H_2_ bubble evolution at high current density (Figure 6h) [50, 51, 52]. EDS analysis revealed a 30% reduction in Ru content with stable Pt levels, indicating continuous Ru leaching. Notably, Fe was detected on the aged cathode (Figures S18 and S19; Table S4), confirming the migration and deposition of anodically dissolved Fe species across the membrane. XPS analysis corroborated the reduction in Ru content without changes in valence state (Figure S20 and Table S5). Finally, the QPS‐PTFE3‐5L HEM was redissolved for ^1^H NMR analysis. The spectra of the aged membrane showed unchanged integral areas for the aryl and α‐carbon hydrogen adjacent to the N^+^ group, with no new peaks in the 4–6 ppm or 8–10 ppm regions (Figure 6i). This confirms the excellent alkali stability of the membrane during long‐term operation, attributable to the intrinsic properties of the HEM and the dilute alkaline feed.

### 2.6 Scale‐Up Demonstration of the HEMEL

**FIGURE 7:**
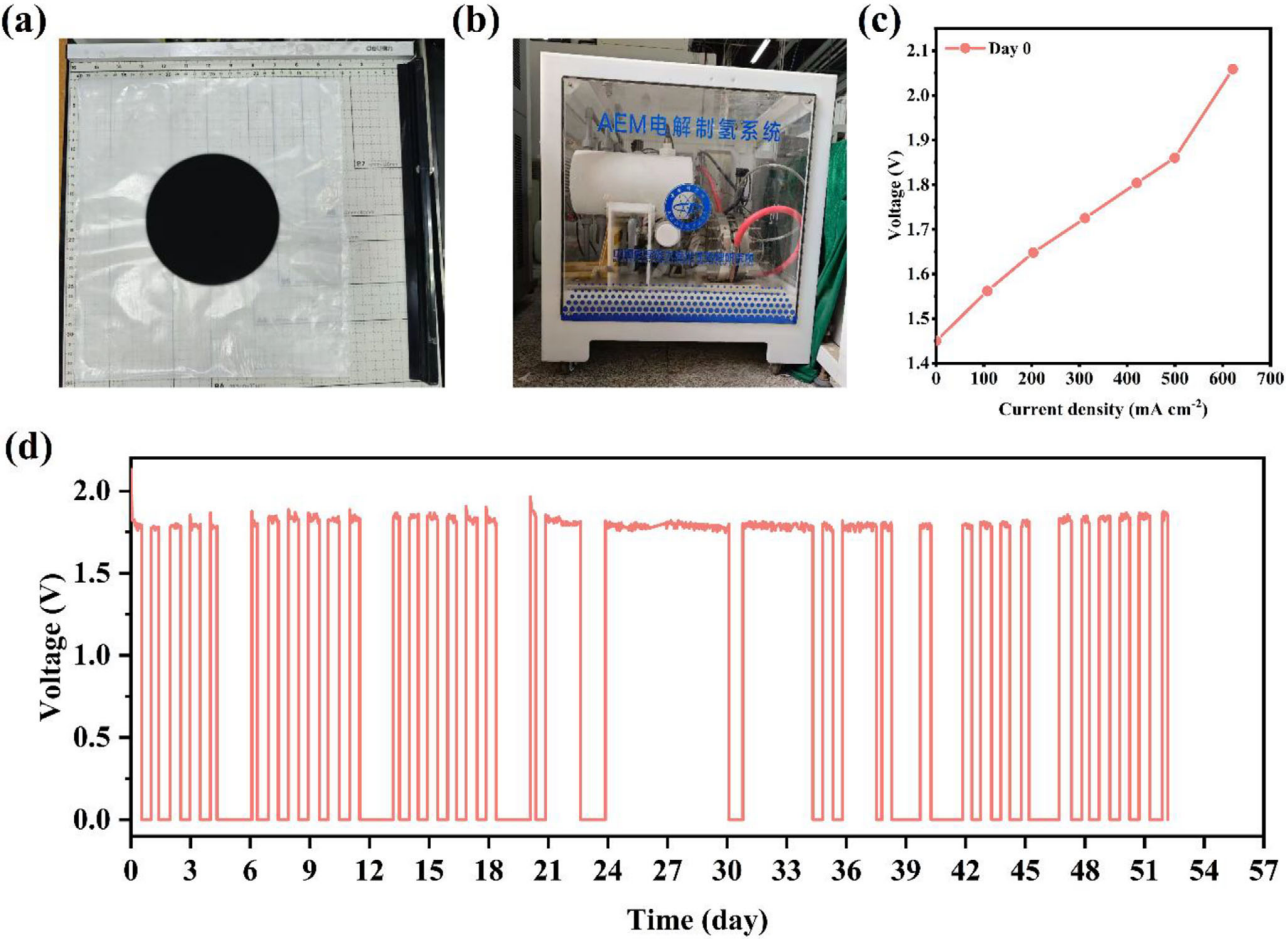
Performance and durability of the scaled 160 cm^2^ HEMEL. Photograph of (a) 160 cm^2^ MEA and (b) integrated single‐cell system. (c) Polarization curve. (d) Voltage stability during intermittent (start‐stop) cycling at 500 mA cm^−2^ in 0.1 m KOH.

To validate the practical scalability and application potential of the membrane design, a large‐area (160 cm^2^) MEA was fabricated using the QPS‐PTFE3‐5L membrane (Figure 7a) and assembled into a single‐cell electrolyzer. The performance of this scaled system was evaluated with 0.1 m KOH feed (Figure 7b). As shown in Figure 7c, it achieved a current density of 550 mA cm^−2^ at 2.0 V. This performance is lower than that of the 5 cm^2^ lab‐scale cell, which can be attributed to the characteristic “scale‐up effect” [22]. To further clarify the origin of this scale‐up effect, the EIS spectra of the 5 and 160 cm^2^ cells were fitted using the equivalent circuit shown in Figure S21, and the fitted parameters are summarized in Table S6. The R_s_ of the 160 cm^2^ cell at Day 0 was 1.8166 Ω cm^2^, approximately 20.8 times higher than that of the 5 cm^2^ cell. The fixture contact resistance of the 160 cm^2^ system was measured to be 0.3247 Ω cm^2^ (Figure S21c). Even after subtracting this contribution, the corrected R_s_ remained 1.4919 Ω cm^2^, still about 17.1 times higher than that of the 5 cm^2^ cell. In addition, R_ct,1_ increased from 0.0294 to 0.5127 Ω cm^2^, corresponding to a 17.4‐fold increase, which is comparable to the increase in R_s_. After 30 days of operation, R_s_ decreased from 1.8166 to 1.2238 Ω cm^2^, while R_ct,1_ decreased from 0.5127 to 0.1794 Ω cm^2^, suggesting partial operational activation, likely arising from improved interfacial wetting, electrolyte penetration, and membrane‐electrode contact during continuous operation. In contrast, R_ct,2_ increased from 0.0843 to 0.6188 Ω cm^2^, indicating that transport limitations became more pronounced during long‐term operation. Overall, these results indicate that the “scale‐up effect” cannot be attributed solely to ohmic resistance, but is also related to interfacial processes and transport limitation [53, 54, 55].

We further assessed the long‐term operational stability of the scaled electrolyzer under start‐stop cycling conditions (Figure 7d). The scaled system demonstrated excellent start‐stop cycling stability, maintaining consistent voltage over repeated daily and random cycles throughout a 52‐day test. Furthermore, it operated stably for 6 consecutive days under a constant current density of 500 mA cm^−2^ without voltage degradation. Over the comprehensive 52‐day test, the cell voltage remained remarkably stable, confirming robust long‐term durability. As an ex situ verification of membrane stability, the membrane recovered after the scaled‐up electrolysis test was characterized by ^1^H NMR. As shown in Figure S22, the characteristic proton signals and their integral ratios remained essentially unchanged compared with those of the pristine membrane, indicating no detectable chemical degradation after operation.

While there is clear scope for optimizing the performance and stability of the scaled system–‐for instance, by engineering the flow fields and improving current distribution–‐the successful fabrication and stable long‐term operation of this 160 cm^2^ HEMEL provide a critical proof‐of‐concept. This work provides a scalable membrane design strategy and a proof‐of‐concept demonstration for the practical development of dilute alkali HEMEL technology.

## 3 Conclusion

In summary, a simple and scalable hot‐pressing protocol was developed for fabricating multilayer PTFE‐reinforced composite HEMs (QPS‐PTFE3‐nL) based on a low‐T_g_ hydroxide exchange polymer. The low‐T_g_ polymer enables excellent interfacial conformability, allowing membranes such as QPS‐PTFE3‐5L to form an optimized MEA interface while retaining high hydration. This was translated into a HEMEL with outstanding performance, delivering 3.7 A cm^−2^ at 1.8 V and exceptional durability–‐operating for over 3300 h at 2.0 A cm^−2^–‐with a dilute 0.1 m KOH feed. Post‐mortem analysis identifies structural collapse and ageing of the CCL as the primary degradation pathway. Furthermore, we successfully fabricated and validated a 160 cm^2^ HEMEL, which demonstrates stable operation for 52 days at 500 mA cm^−2^, including excellent start‐stop cycling tolerance. This work provides a practical and scalable HEM design strategy, advancing the development of durable, high‐performance HEMEL systems for renewable energy integration and industrial applications.

## Funding

Strategic Priority Research Program of the Chinese Academy of Sciences, No. XDA0400301; National Natural Science Foundation of China, No. 22279134; LiaoNing Revitalization Talents Program, No. XLYC2203150; Liaoning Binhai Laboratory, LBLG‐2025‐01.

## Conflicts of Interest

The authors declare no competing interests.

## Supporting information




**Supporting File**: advs77388‐sup‐0001‐SuppMat.docx.

## Data Availability

The data that support the findings of this study are available from the corresponding author upon reasonable request.
