# Supplementary material for: Scalable Multilayer PTFE‐Reinforced Membranes for Durable, High‐Current‐Density Hydroxide Exchange Membrane Water Electrolysis in Dilute Alkali
Source: Adv Sci (Weinh). 2026 Aug 24;13(63):e77388. doi: 10.1002/advs.77388 (PMC13502344; doi:10.1002/advs.77388)
Supplement: Supplementary file 1 [file ADVS-13-e77388-s001.docx]

**Scalable Multilayer PTFE-Reinforced Membranes for Durable, High-Current-Density Hydroxide Exchange Membrane Water Electrolysis in Dilute Alkali**

Zhiwei Ren, Yun Zhao*, Rui Zhang, Tao Wei, Yingran Zhu, Yangkai Han, Tongzhou Li, Haitao Zhang, Zhigang Shao*

Experimental Section/Methods

*Materials*: Polystyrene (average M.W. 260,000) was purchased from Thermo Fisher Scientific Inc. 6-bromohexanoyl chloride, trifluoroacetic acid (TFA), dichloromethane (CH_2_Cl_2_) and triethylsilane were purchased from Aladdin Chemical Co., Ltd. Dimethyl sulfoxide (DMSO), N-Methyl-2-pyrrolidone (NMP), quinuclidine, ethanol and ethyl acetate were purchased from Shanghai Acmec Biochemical Technology Co., Ltd. Potassium bromide (KBr), Aluminum chloride (AlCl_3_), sodium bicarbonate (NaHCO_3_), potassium hydroxide (KOH), sodium chloride (NaCl), sodium nitrate (NaNO_3_) and silver nitrate (AgNO_3_) were purchased from Sinopharm Chemical Reagent Co., Ltd. All chemicals were used without further purification. Deionized water (conductivity:18.2 MΩ cm^-1^) was used in all the tests.

*Synthesis of PSHOBr*: The PSHOBr polymer was synthesized via Friedel-Crafts acylation reaction. In a glove box, 10 g of PS was dissolved in CH_2_Cl_2_ in a flask. Subsequently, 8.2 g of 6-bromohexanoyl chloride and a determined amount of AlCl_3_ were added. The reaction mixture was stirred at 25 °C for 15 hours. The product was precipitated by adding the mixture into 500 mL of ethanol, collected by filtration, and washed three times with ethanol. The resulting white solid was vacuum-dried at 30 °C for 24 hours, yielding PSHOBr with a 91.6% yield.

*Synthesis of PSHBr*: PSHBr was prepared via a silane-mediated reduction of the carbonyl group in PSHOBr. In a 1000 mL flask, 14 g of PSHOBr was dissolved in CH_2_Cl_2_. TFA and triethylsilane were added, and the reaction was stirred at 25 °C for 48 h. The mixture was then quenched with ice-cold 2 M KOH and washed with deionized water until the aqueous phase was neutral. The organic layer was precipitated into 500 mL of ethanol. The resulting solid was collected by filtration, washed with ethanol, and dried under vacuum at 30 °C for 24 h to yield PSHBr as a fibrous white solid (91% yield).

*Synthesis of QPS*: QPS was prepared by quaternizing PSHBr with quinuclidine in a Menshutkin reaction. 1 g of PSHBr was dissolved in NMP at 80 °C, followed by the addition of quinuclidine (0.45 g). The reaction was stirred at 80 °C for 24 h. The cooled mixture was concentrated via reduced pressure distillation, precipitated into ethyl acetate, filtered, and washed with ethyl acetate. Drying under vacuum at 60 °C for 24 h afforded QPS as a white solid (97% yield).

*Preparation of QPS-PTFE3-nL (n = 3, 4, 5) composite HEMs*: Multilayer membranes QPS-PTFE3-nL (n = 3, 4, 5) were prepared by hot-pressing single-layer membranes (QPS-PTFE3-1L), which was fabricated by doctor-blade coating a 5 wt% QPS ethanol solution onto a 3 μm thick porous PTFE substrate, followed by air drying. For multilayer assembly, the required number of QPS-PTFE3-1L layers were stacked and hot-pressed at 110 °C under 3 MPa for 30 minutes per lamination step. For instance, QPS-PTFE3-3L was prepared by first laminating two single layers to form a bilayer, which was then laminated with a third single layer.

*Characterization of chemical structure and morphology*: The chemical composition and the structure of HEMs were characterized by ^1^H nuclear magnetic resonance (^1^H NMR, AVANCE III 400 MHz). DMSO-d_6_ and CDCl_3_ were used as the solvents for ^1^H NMR. The 5% TFA was added as the co-solvent to avoid the effect of H_2_O at 3.33 ppm. The morphologies of the surface and cross-section of HEMs in Br^-^ form were observed using a scanning electron microscope (SEM, Hitachi Regulus 8100). The aggregation state and the microphase separation structure for wet-state OH^-^-form HEMs were studied by small-angle X-ray scattering (SAXS, Xenocs Xeuss 2.0/3.0). Hydrogen permeation tests of wet membranes were conducted with reference to previous literature.^[1]^ The hydrogen flow rate was 100 sccm, and the Ar purge gas flow rate was 15 sccm.

*Ion exchange capacity (IEC)*: The IECs of the HEMs in Cl^-^ form were measured by the Mohr titration. The dry HEMs in Cl^-^ form were weighed (W_dry_) and soaked in 40 ml of 0.5 M NaNO_3_ solution at 80 °C twice (each time for 12 h). Then, an automatic potentiometric Titrator (ZDJ-4B) was used for the titration with a 0.01 M AgNO_3_ solution. The Ag/AgCl reference electrode and ion-selective electrode were used to monitor the titration for determining the volume (V_AgNO3_) of 0.01M AgNO_3_ consumed. The IEC (mmol g^-1^) is based on equation (1):

$\text{IEC}\text{ }\text{=}\text{ }\frac{\text{0.01*}\text{V}_{\text{AgNO}_{\text{3}}}}{\text{W}_{\text{dry}}}$	(1)

*Ionic conductivity (σ)*: The ionic conductivities of HEMs were measured over a frequency range of 1-100 kHz by a four-probe method using an electrochemical workstation (Gamry Interface 1000E). The rectangular sample in OH^-^ form was placed on four platinum electrodes in the homemade test fixture. The AC impedances of the sample at different temperatures were recorded in DI water. The ionic conductivity (σ, mS cm^-1^) can be calculated based on the following equation (2):

$\text{σ}\text{ }\text{=}\text{ }\frac{\text{L}}{\text{AR}}$	(2)

L (cm) is the distance between the two platinum electrodes, A (cm^2^) stands for the cross-sectional area of the membrane, and R (kΩ) represents the impedance of the sample.

*Water uptake and swelling ratio*: First, the HEMs in OH^-^ form were dried in a vacuum oven at 80 ℃ for 24 h with the weight (W_dry_) and length (L_dry_) recorded. The HEMs were swollen in deionized water for 24 h over the temperature range of 30-80 °C. After that, the HEMs were quickly wiped off the surface water with the weight (W_wet_) and length (L_wet_) recorded. The water uptake (WU, %) and the swelling ratio (SR, %) were calculated by the following equation (3) and equation (4):

$\text{WU}\text{ }\text{= }\frac{\text{W}_{\text{wet}}\text{-}\text{W}_{\text{dry}}}{\text{W}_{\text{dry}}}\text{×100\%}$	(3)

$\text{SR}\text{ }\text{= }\frac{\text{L}_{\text{wet}}\text{-}\text{L}_{\text{dry}}}{\text{L}_{\text{dry}}}\text{×100\%}$	(4)

*Mechanical properties and thermal stability*: The mechanical properties of the HEMs in Br^-^ were tested by a universal testing machine (WDW-10). Typically, the HEMs were cut into rectangular specimens with dimensions of 1×5 cm using a standard mold. The tensile strength and the elongation at break for HEMs were measured under ambient conditions at a stretching rate of 2 mm min^-1^. The thermal stability of the HEMs in Br^-^ form was measured using a thermo-gravimetric analysis instrument (TG-DSC, STA 449 F3 Jupiter) under N_2_ atmosphere from 50 to 800 ℃ at the ramping rate of 10 °C min^-1^.

*Alkali stability*: The alkali stability of HEMs was investigated by monitoring the changes in IEC and HCO_3_^-^ conductivity (at 50 °C) with aging time. Three samples were prepared for each HEM for testing. The degradation was conducted in 5 M KOH at 50 °C. Prior to testing, the initial IEC (IEC_0_) and HCO_3_^-^ conductivity (σ_0_) of the samples were characterized. Subsequently, the samples were taken out at specific time intervals to record their IEC (IEC_t_) and HCO_3_^-^ conductivity (σ_t_). The alkali stability and structural stability of the HEMs were calculated using equation (5) and equation (6):

$\text{IEC remaining }\text{(}\text{\%}\text{)}\text{ }\text{=}\text{ }\frac{\text{IEC}_{\text{t}}}{\text{IEC}_{\text{0}}}\text{×100\%}$	(5)

$\text{HCO}_{\text{3}}^{\text{-}}\text{ conductivity remaining }\text{(\%)}\text{ }\text{=}\text{ }\frac{\text{σ}_{\text{t}}}{\text{σ}_{\text{0}}}\text{×100\%}$	(6)

During the testing process, the samples continued to swelling. Based on previous conclusions, the swelling primarily occurred in the through-plane direction. Thus, the thickness change rate (Equation (7)) was used to independently evaluate the structural stability of the samples:

$\text{Swelling ratio}_{\text{through-plane}}\text{ (\%) }\text{=}\text{ }\frac{\text{L}_{\text{t}}}{\text{L}_{\text{0}}}\text{×100\%}$	(7)

*HEMEL performance and durability*: A MEA with a 5 cm^2^ active area was constructed using the QPS-PTFE3-5L HEM. The anode consisted of a homemade γ-Ni(Fe)OOH@NF catalyst.^[2]^ For the cathode, a catalyst ink was prepared by dispersing commercial PtRu/C (40 wt% Pt, 20 wt% Ru, JM) and QPS ionomer in an isopropyl/water solvent via ultrasonication for one hour at 15 °C. The ink was spray-coated on the membrane in its Br^-^ form to achieve a metal loading of 1.0 mg cm^-2^. The cathode-coated membrane was then converted to the OH^-^ form by immersion in 1 M KOH at 80 °C for 24 hours, with the KOH solution refreshed every 8 hours. The final MEA was assembled by hot-pressing the membrane between the anode and cathode.

The HEMEL performance was evaluated in pure water, 0.1 M KOH and 1 M KOH respectively. All measurements were conducted using a Gamry Interface 1000E potentiostat integrated with a custom test station. Prior to testing, each cell was activated by operating at 100 mA cm^-2^ for 10 minutes and 300 mA cm^-2^ for 30 minutes. Linear sweep voltammetry (LSV) was performed by scanning the current density at a rate of 20 mA cm^-2^ s^-1^ up to 2 V. Electrochemical impedance spectroscopy (EIS) was measured at 100 mA cm^-2^ with a 20 mA cm^-2^ amplitude over a frequency range from 100 kHz to 1 Hz. The cell was then operated at 100 mA cm^-2^ until a stable voltage was reached following each electrolyte change, after which data collection began. Long-term durability was assessed at 50 °C by operating the cell at 2.0 A cm^-2^ with a continuous feed of 0.1 M KOH to the anode. Polarization curves and EIS data were recorded before and after this stability test.

For detailed electrochemical analysis, LSV was also performed from 1.0 to 2.2 V (vs. RHE) at a scan rate of 5 mV s^-1^ using a Hg/HgO reference electrode. Morphological changes were observed by SEM (Hitachi Regulus 8100) and AFM (Bruker Dimension Icon). Elemental distribution and content variations were characterized by EDX (Hitachi Regulus 8100). Valence state changes were analyzed by XPS (Thermo Scientific K-Alpha). Particle size distribution was determined by HR-TEM (JEOL JEM-2100F). Chemical structure changes of the membrane were investigated by 1H NMR (AVANCE III 400 MHz) spectroscopy.

**Figure S1**. Optical images of QPS-PTFE3-1L.

**Figure S2**. (a) Optical photograph and (b) cross-sectional SEM images of five resin-unfilled PTFE substrates after hot-pressing treatment.

**Figure S3**. Wet-state H_2_ permeability comparison of QPS-PTFE3-5L and commercial PiperION A20 membranes.

**Figure S4**. Swelling ratio of QPS and QPS-PTFE3-nL (n = 3, 4, 5) HEMs in (a) through-plane and (b) in-plane directions.

**Figure S5**. Comparison of OH^-^ conductivity of QPS, QPS-PTFE3-nL and commercial PiperION A20 membranes.

**Figure S6**. Potential degradation pathways of QPS polyelectrolyte.

**Figure S7**. The alkali stability and structural stability of the QPS-PTFE3-nL (n = 3, 4, 5) HEMs. (a) HCO_3_^-^ conductivity remaining and (b) through-plane swelling ratio of HEMs in 5 M KOH at 50 °C.

**Figure S8**. HEMEL performance of QPS-PTFE3-nL.

**Figure S9**. Cross-sectional SEM images of the MEA anode side: (a) with hot pressing and (b) without hot pressing.

**Figure S10**. HEMEL performance comparison of QPS-PTFE3-5L and commercial PiperION A20 membranes at 60 °C in 0.1 M KOH: (a) polarization curves and corresponding H_2_ concentration in the O_2_ outlet; (b) Nyquist plots.

**Figure S11**. Morphology images of (a) pristine and (b) aged γ-Ni(Fe)OOH@NF.

**Figure S12**. EDS spectra of (a) pristine and (b) aged γ-Ni(Fe)OOH@NF.

**Figure S13**. Elemental distribution maps of (a, b, c) pristine and (d, e, f) aged γ-Ni(Fe)OOH@NF.

**Figure S14**. XPS spectra of pristine and aged γ-Ni(Fe)OOH@NF.

**Figure S15**. Morphology images of (a) pristine and (b) aged CCL.

**Figure S16**. AFM images of (a) pristine and (b) aged CCL.

**Figure S17**. Morphology images of (a) pristine and (b) aged PtRu/C catalyst.

**Figure S18**. EDS spectra of (a) pristine and (b) aged PtRu/C.

**Figure S19**. Elemental distribution maps of (a-d) pristine and (e-i) aged PtRu/C.

**Figure S20**. XPS spectra of pristine and aged PtRu/C.

**Figure S21**. (a) Polarization curve, (b) Nyquist plots and (c) contact resistance of 160 cm^2^ single-cell HEMEL system.

**Figure S22.** ^1^H NMR analysis of the membrane after the scaled-up electrolysis test.

**Table S1**. Comparison of EIS equivalent circuit fitting parameters of HEMEL under different feed conditions.

|  | Pure water | 0.1 M KOH | 1.0 M KOH |
| --- | --- | --- | --- |
| R_s_ (Ω cm^2^) | 0.1911 | 0.0872 | 0.0523 |
| CPE_1_-T (Ω ^-1^ cm^-2^ s^n^) | 0.0039 | 0.4556 | 0.6418 |
| CPE_1_-P | 0.8820 | 0.6294 | 0.9863 |
| R_ct,1_ (Ω cm^2^) | 0.0740 | 0.0294 | 0.1218 |
| CPE_2_-T (Ω ^-1^ cm^-2^ s^n^) | 0.1733 | 0.3326 | 1.3716 |
| CPE_2_-P | 0.5294 | 0.8149 | 0.6583 |
| R_ct-2_ (Ω cm^2^) | 0.6017 | 0.3037 | 0.2004 |

**Table S2.** Fitted EIS parameters of HEMEL cells assembled with QPS-PTFE3-5L and PiperION A20 membranes at 60 °C in 0.1 M KOH.

|  | QPS-PTFE3-5L | PiperION A20 |
| --- | --- | --- |
| R_s_ (Ω cm^2^) | 0.0671 | 0.0765 |
| R_ct,1_ (Ω cm^2^) | 0.0596 | 0.0817 |
| R_ct-2_ (Ω cm^2^) | 0.0120 | 0.0106 |

**Table S3**. Performance and durability of currently reported state-of-the-art HEMEL.

| HEM | Anode | Cathode | Electrolyte  (M) | Temp  (°C) | Performance  (mA cm^-2^, @1.8V) | Durability | Ref. |
| --- | --- | --- | --- | --- | --- | --- | --- |
| QPS-PTFE3-5L | γ-Ni(Fe)OOH@NF | PtRu/C | 0.1  (KOH) | 50 | 3700 | 3360 h@2000 mA cm^-2^ (37.5 μV h^-1^) | This work |
| PiperION A40 | NiFe LDH | IrRu/C | 0.05  (KOH) | 60 | 1280 | 1040 h@1000 mA cm^-2^ (74.2 μV h^-1^) | [3] |
| HTMA-DAPP | NiFe@NF | PtRu/C | 0.1  (NaOH) | 60 | 3200 | N.A. | [4] |
| QPPO | NiCo_2_O_4_ | Pt/C | 0.1  (NaOH) | 40 | 212 | N.A. | [5] |
| PAP-TP-85-MQN-10C | IrO_x_ | Pt/C | 0.1  (KOH) | 60 | 480 | N.A. | [6] |
| Mg-Al LDHs | CuCoO_3_ | Ni/(CeO_2_-La_2_O_3_)/C | 0.1  (NaOH) | 60 | 56 | 600 h@80 mA cm^-2^ (130.0 μV h^-1^) | [7] |
| QPC-TMA | eCCO | Pt/C | 0.1  (KOH) | 45 | 870 | 3000 h@500 mA cm^-2^ (11.3 μV h^-1^) | [8] |
| X37-50T | Cu_0.5_Co_2.5_O_4_ | Ni-LCO/C | 0.1  (KOH) | 45 | 450 | 100 h@500 mA cm^-2^ (32.0 μV h^-1^) | [9] |
| Fumapem-3-PE-30 | NiCoFe | Ni–MoO_2_ | 0.1  (KOH) | 50 | 260 | 65 h@500 mA cm^-2^ | [10] |
| HTMA-DAPP | IrO_2_ | PtRu/C | 0.1  (NaOH) | 60 | 2000 | 24 h@200 mA cm^-2^ (4.16 mV h^-1^) | [11] |
| HMT-PMBI | Ir | Pt/C | 0.1  (KOH) | 60 | 700 | 100 h@1000 mA cm^-2^ | [12] |
| GT-75-5 | LSC | NiMo-a | 0.3  (KOH) | 60 | 275 | 60 h@1000 mA cm^-2^ | [13] |
| LDPE-VBC-TMA | S-HEO | Pt/C | 0.1  (KOH) | 60 | 680 | N.A. | [14] |
| PFPBPF-QA | Co_3_O_4_ | Pt/C | 0.2  (KOH) | 50 | 1430 | 800 h@1000 mA cm^-2^ (2.0 μV h^-1^) | [15] |
| PiperION | Co_3_O_4_ | Pt/C | 0.2  (KOH) | 50 | 1160 | 800 h@1000 mA cm^-2^ (30.0 μV h^-1^) | [15] |
| AF1-HNN8-50 | Ir | Pt/C | 0.1  (KOH) | 50 | 690 | 16.7 h@500 mA cm^-2^ (3.21 mV h^-1^) | [16] |
| PiperION A80 | Co_3_O_4_ | Pt/C | 0.1  (KOH) | 80 | 900 | 640 h@2000 mA cm^-2^ (5.0 μV h^-1^) | [17] |
| Z-S-20 | NiFe | NiMo | 0.1  (KOH) | 80 | 920 | N.A. | [18] |
| T3-1.0-0.5 | NiFe | NiMo | 1.0  (KOH) | 60 | 3300 | 500 h@2000 mA cm^-2^ (77.0 μV h^-1^) | [19] |
| PAMP | NiFe-LDH/NF | Ni_4_Mo/MoO_2_/NF | 1.0  (KOH) | 80 | 4200 | 1473 h@1000 mA cm^-2^ (31.0 μV h^-1^) | [20] |
| Men-PF | NiFe-LDH | Raney Ni | 5 wt%  (KOH) | 60 | 4050 | 800 h@1000 mA cm^-2^ | [21] |
| HQPC-TMA | IrO_2_ | PtRu/C | 1.0  (KOH) | 80 | 7400 | 1000 h@1000 mA cm^-2^ (79.0 μV h^-1^, 60 °C) | [22] |
| PFTP-13 | IrO_2_ | Pt/C | 1.0  (KOH) | 60 | 2400 | 1100 h@500 mA cm^-2^ | [23] |

**Table S4**. Elemental content variations of anode and cathode catalyst layers after long-term durability test (determined by EDS analysis).

| Anode: γ-Ni(Fe)OOH@NF | | | | |
| --- | --- | --- | --- | --- |
|  | Pristine | | Aged | |
| Element | Weight Percent  (Wt. %) | Atomic Percent  (At. %) | Weight Percent  (Wt. %) | Atomic Percent  (At. %) |
| Ni | 63.98 | 36.13 | 71.16 | 42.47 |
| Fe | 6.94 | 4.12 | 3.14 | 1.97 |
| O | 28.81 | 59.71 | 25.34 | 55.49 |
| Pt | 0.27 | 0.05 | 0.36 | 0.06 |
| Ru | 0.00 | 0.00 | 0.00 | 0.00 |

| Cathode: PtRu/C | | | | |
| --- | --- | --- | --- | --- |
|  | Pristine | | Aged | |
| Element | Weight Percent  (Wt. %) | Atomic Percent  (At. %) | Weight Percent  (Wt. %) | Atomic Percent  (At. %) |
| Pt | 30.71 | 3.33 | 32.28 | 3.40 |
| Ru | 15.29 | 3.20 | 10.13 | 2.06 |
| C | 50.11 | 88.31 | 50.77 | 86.76 |
| Ni | 0.00 | 0.00 | 0.00 | 0.00 |
| Fe | 0.00 | 0.00 | 1.05 | 0.39 |
| O | 3.89 | 5.15 | 5.77 | 7.40 |

**Table S5.** Elemental content variations of anode and cathode catalyst layers after long-term durability test (determined by XPS analysis).

|  | Anode |  |  | Cathode |  |
| --- | --- | --- | --- | --- | --- |
| Element | Pristine  (At. %) | Aged  (At. %) | Element | Pristine  (At. %) | Aged  (At. %) |
| Ni | 21.08 | 20.24 | Pt | 58.09 | 64.9 |
| Fe | 15.08 | 8.49 | Ru | 41.91 | 35.1 |
| O | 63.84 | 71.27 |  |  |  |

**Table S6.** Comparison of EIS equivalent circuit fitting parameters for 5 cm^2^ and 160 cm^2^ HEMEL single cells with 0.1 M KOH.

|  | 5 cm^2^ | 160 cm^2^ | |
| --- | --- | --- | --- |
|  |  | Day 0 | Day 30 |
| R_s_ (Ω cm^2^) | 0.0872 | 1.8166 | 1.2238 |
| R_ct,1_ (Ω cm^2^) | 0.0294 | 0.5127 | 0.1794 |
| R_ct-2_ (Ω cm^2^) | 0.3037 | 0.0843 | 0.6188 |

**References**

1.	C. Zhu, Y. Peng, K. Li, L. Liu, W. Yang, Journal of Membrane Science 2024, 709, https://doi.org/10.1016/j.memsci.2024.123140.

2.	J. Chi, H. Yu, B. Qin, L. Fu, J. Jia, B. Yi, Z. Shao, *ACS Applied Materials & Interfaces* 2016, 9 (1), 464, https://doi.org/10.1021/acsami.6b13360

3.	H. Shen, F. Y. Gao, H. Li, J. Xu, M. Jaroniec, Y. Zheng, S. Z. Qiao, *J Am Chem Soc* 2025, 147 (26), 22677, https://doi.org/10.1021/jacs.5c04194

4.	D. Li, E. J. Park, W. Zhu, Q. Shi, Y. Zhou, H. Tian, Y. Lin, A. Serov, B. Zulevi, E. D. Baca, C. Fujimoto, H. T. Chung, Y. S. Kim, *Nature Energy* 2020, 5 (5), 378, https://doi.org/10.1038/s41560-020-0577-x

5.	Z. Feng, P. O. Esteban, G. Gupta, D. A. Fulton, M. Mamlouk, *International Journal of Hydrogen Energy* 2021, 46 (75), 37137, https://doi.org/10.1016/j.ijhydene.2021.09.014

6.	Q. Sun, N. J. Oliveira, S. Kwon, S. Tyukhtenko, J. J. Guo, N. Myrthil, S. A. Lopez, I. Kendrick, S. Mukerjee, L. Ma, S. N. Ehrlich, J. Li, W. A. Goddard, Y. Yan, Q. Jia, *Nature Energy* 2023, 8 (8), 859, https://doi.org/10.1038/s41560-023-01302-y

7.	L. Zeng, T. S. Zhao, *Nano Energy* 2015, 11, 110, https://doi.org/10.1016/j.nanoen.2014.10.019

8.	M. J. Jang, S. H. Yang, M. G. Park, J. Jeong, M. S. Cha, S.-H. Shin, K. H. Lee, Z. Bai, Z. Chen, J. Y. Lee, S. M. Choi, *ACS Energy Letters* 2022, 7 (8), 2576, https://doi.org/10.1021/acsenergylett.2c01049

9.	M. J. Jang, J. Yang, J. Jeong, G. H. Kim, C. Y. Kwon, N. V. Myung, K. H. Lee, S. M. Choi, *ACS Sustainable Chemistry & Engineering* 2021, 9 (37), 12508, https://doi.org/10.1021/acssuschemeng.1c03715

10.	A. Y. Faid, A. O. Barnett, F. Seland, S. Sunde, *ACS Appl Energy Mater* 2021, 4 (4), 3327, https://doi.org/10.1021/acsaem.0c03072

11.	K. Li, S. Yu, D. Li, L. Ding, W. Wang, Z. Xie, E. J. Park, C. Fujimoto, D. A. Cullen, Y. S. Kim, F. Y. Zhang, *ACS Appl Mater Interfaces* 2021, 13 (43), 50957, https://doi.org/10.1021/acsami.1c14693

12.	B. Y. Chen, P. Mardle, S. Holdcroft, *Journal of Power Sources* 2022, 550, 232134, https://doi.org/ARTN 23213410.1016/j.jpowsour.2022.232134.

13.	M. Amirsalehi, N. Ul Hassan, V. S. S. Mosali, I. Street, M. Manjum, S. A. Lateef, J. Bohannon, S. Mckinney, A. G. Divekar, P. A. Kohl, W. E. Mustain, *Appl Catal B-Environ* 2025, 378, 125558, https://doi.org/ARTN 12555810.1016/j.apcatb.2025.125558

14.	S. C. Karthikeyan, S. Ramakrishnan, S. Prabhakaran, M. R. Subramaniam, M. Mamlouk, D. H. Kim, D. J. Yoo, *Small* 2024, 20 (45), e2402241, https://doi.org/10.1002/smll.202402241

15.	H. Lim, J. Y. Jeong, G. Shin, C. Kim, G. Choi, S. W. Myeong, S. M. Choi, T. Park, *Advanced Energy Materials* 2024, 14 (34), 2401725, https://doi.org/10.1002/aenm.202401725

16.	P. Fortin, T. Khoza, X. Cao, S. Y. Martinsen, A. Oyarce Barnett, S. Holdcroft, *Journal of Power Sources* 2020, 451, 227814, https://doi.org/10.1016/j.jpowsour.2020.227814

17.	A. W. Tricker, T. Y. Ertugrul, J. K. Lee, J. R. Shin, W. Choi, D. I. Kushner, G. Wang, J. Lang, I. V. Zenyuk, A. Z. Weber, X. Peng, *Advanced Energy Materials* 2023, 14 (9), https://doi.org/10.1002/aenm.202303629

18.	W. Zheng, L. He, T. Tang, R. Ren, H. Lee, G. Ding, L. Wang, L. Sun, *Angew Chem Int Ed Engl* 2024, 63 (34), e202405738, https://doi.org/10.1002/anie.202405738

19.	T. Tang, H. Lee, Z. Wang, Z. Li, L. Wang, D. Chen, W. Zheng, Q. Liu, L. He, G. Ding, Z. Tian, L. Sun, *Energy & Environmental Science* 2024, 17 (20), 7816, https://doi.org/10.1039/d4ee02428a

20.	L. Yin, R. Ren, L. He, H. Lee, Q. Zhang, G. Ding, L. Wang, L. Sun, *Angew Chem Int Ed Engl* 2025, 64 (19), e202503715, https://doi.org/10.1002/anie.202503715

21.	H. Kim, S. Jeon, J. Choi, Y. S. Park, S. J. Park, M. S. Lee, Y. Nam, H. Park, M. Kim, C. Lee, S. E. An, J. Jung, S. Kim, J. F. Kim, H. S. Cho, A. S. Lee, J. H. Lee, *ACS Nano* 2024, 18 (47), 32694, https://doi.org/10.1021/acsnano.4c10212

22.	S. Kim, S. H. Yang, S.-H. Shin, H. J. Cho, J. K. Jang, T. H. Kim, S.-G. Oh, T.-H. Kim, H. Han, J. Y. Lee, *Energy & Environmental Science* 2024, 17 (15), 5399, https://doi.org/10.1039/d4ee01003e

23.	N. Chen, S. Y. Paek, J. Y. Lee, J. H. Park, S. Y. Lee, Y. M. Lee, *Energy & Environmental Science* 2021, 14 (12), 6338, https://doi.org/10.1039/d1ee02642a
